# Interplay between reactive oxygen species and autophagy in the course of age-related macular degeneration

**DOI:** 10.17179/excli2020-2915

**Published:** 2020-09-25

**Authors:** Malgorzata Nita, Andrzej Grzybowski

**Affiliations:** 1 Domestic and Specialized Medicine Centre "Dilmed" Katowice, Poland; 2 Department of Ophthalmolgy, Medical Faculty, University of Warmia and Mazury, Olsztyn, Poland; 3 Institute for Research in Ophthalmology, Poznań, Poland

**Keywords:** reactive oxygen species, oxidative stress, autophagy, age-related macular degeneration, retina disease, ophthalmology

## Abstract

Pathological biomolecules such as lipofuscin, methylglyoxal-modified proteins (the major precursors of advanced glycationend products), misfolding protein deposits and dysfunctional mitochondria are source of oxidative stress and act as strong autophagic stimulators in age-related macular degeneration. Disturbed autophagy accelerates progression of the disease, since it leads to retinal cells' death and activates inflammation by the interplay with the NLRP3 inflammasome complex. Vascular dysfunction and hypoxia, as well as circulating autoantibodies against autophagy regulators (anti-S100A9, anti-ANXA5, and anti-HSPA8, A9 and B4) compromise an autophagy-mediated mechanism as well. Metformin, the autophagic stimulator, may act as a senostatic drug to inhibit the senescent phenotype in the age-related macular degeneration. PGC-1α , Sirt1 and AMPK represent new therapeutic targets for interventions in this disease.

## Introduction

Age-related macular degeneration (AMD) is the leading cause of irreversible blindness in patients aged 65 years and above of industrialized countries (Bourne et al., 2013[12]), caused by the interplay between polygenic background and multiple acquired factors (Chakravarthy et al., 2010[18]). Genetic predisposition and epigenetic modifications together with advanced age and its related physiological cell apoptosis and tissue involution are the strongest risk factors of this multifactorial disease, however important are also moderate, non modifable risk factors such as family history of AMD or sex, and numerous environmental, modifiable influences (Chakravarthy et al., 2010[18]). Abnormal immunologic events and inflammatory responses play a crucial role in AMD etiopathogenesis as well (Parmeggiani et al., 2012[91]), but AMD is not a classical inflammatory disease per se (Nita et al., 2014[85]; Parmeggiani et al., 2012[91]; Sparrow et al., 2012[107]). The pathological changes associated with AMD affect the anatomically and functionally tissue complex of the central part of the retina (macula) including photoreceptors, retinal pigment epithelium (RPE) cells, extracellular matrix (Bruch's membrane, BrM) and choriocapillaries (Jong, 2006[55]; Nita et al., 2014[86]). Soft drusen (SD) and/or pigmentary abnormalities visible clinically characterize slowly and progressively arising early AMD form. Visual loss in the course of advanced AMD is caused by the “geographic“ atrophic death of photoreceptors, RPE cells and choriocapillaries; so called “dry AMD”, GA/AMD, or by formation of the choroidalneovascular (CNV) membrane as a result of pathological angiogenesis; so called “exudative AMD”, CNV/AMD (Jong, 2006[55]). 

During the course of aging, accumulation of the reactive oxygen species (ROS) leads to oxidative stress (OS) in RPE cells vulnerable to oxidative injury. In majority of cases OS is a primary source of excess ROS, which impairs RPE organelle function and evokes functional abnormalities, contributes to altering biomolecules and protein stability, triggers protein misfolding and formation of protein aggregates (aggresomes), induces inflammatory cascades in aging RPE cells, and leads to oxidative stress-mediated senescence and death of RPE cells and photoreceptors (they dye subsequently to the death of RPE) observed in the advanced stages of AMD (Blasiak et al., 2014[7]). The pathogenic role of ROS overproduction in the pathogenesis of AMD is convincingly supported by clinical studies showing that supplementation with antioxidants can slow down progression of this disease (Moriarty-Craige et al., 2005[82]). 

Autophagy is one of the most important cellular clearance systems for degrading and removing organelles and aggregated proteins impaired by oxidation (Blasiak et al., 2014[7]). This catabolic pathway acts as strong antioxidant and cytoprotective cellular response aimed to counteract oxidative stressful and oxidative damaging conditions (Blasiak et al., 2014[7]). Dysregulated autophagy in the RPE cells, caused by hyperactivity of ROS, may lead to increased cell susceptibility to OS and detrimental protein accumulation (Blasiak et al., 2014[7]). Laboratory studies implicate that dysfunctional autophagy is an underlying mechanism contributing to the pathophysiology of AMD. The increased contents of autophagy markers, i.e. autophagy related 5 (Atg5) protein and LC3 protein, was evidenced in drusen of cadaver eyes from old patients suffering from AMD (Wang et al., 2009[123]). The conversion of soluble LC3-I to lipid bound LC3-II is associated with the formation of autophagosomes (see below). The measurement of the ratio of LC3-I/ LC3-II; a standard marker for autophagosomes, revealed impaired autophagy in AMD RPE as compared with normal RPE (Golestaneh et al., 2017[40]). 

Autophagic flux was reduced in AMD RPE as compared with normal RPE, as shown by inability of AMD RPE to downregulate the ubiquitin-binding protein p62 levels during starvation; p62 also known as sequestosome-1 and encoded by the SQSTM1 gene is an autophagosome cargo protein that targets other proteins that bind to it for selective autophagy, see below). Impaired autophagic pathway confirmed also analysis of the late autophagic vesicles; immunostaining with lysosome-associated membrane protein 1 (LAMP-1) antibody revealed enlarged and annular LAMP-1-positive organelles in AMD RPE as opposed to the smaller discrete puncta observed in normal RPE (Golestaneh et al., 2017[40]).

Since, the multiple papers from the recent years underlie both: the role of oxidative stress as well as autophagic pathway in the AMD pathogenesis, we have decided to review and analyze their interplay in the course of this illness. 

The scientific literature dated from 2010 to June 2020 was examined thoroughly with the use of Medline and PubMed. Publications dated from 2015 to 2020 were analyzed in detail, using search terms such as above-mentioned keywords.

## The General Characteristic of the Reactive Oxygen Species, Oxidative Stress and Autophagy

The reactive oxygen species (ROS) are formed under physiological conditions due to the partial reduction of molecular oxygen. Superoxide anion (O2-), hydroxyl radical (OH∙), hydrogen peroxide (H2O2), and singlet oxygen (1O2) arise mainly as a product of the respiratory chain in cellular mitochondrion and as a result of the exposure to UV light, in photochemical and enzymatic reactions, ionizing radiation or heavy metal ions (Dröge, 2002[26]).

Low levels of ROS production are necessary to maintain physiological cell functions (proliferation, host defense, signal transduction and gene expression) (Dröge, 2002[26]). Eucariotic cells have several intracellular defense mechanisms providing balance between ROS generation and clearance. Under physiological conditions, many enzymatic antioxidants (Cu/Zn-superoxide dismutase in the cytosol, manganese superoxide dismutase in the mitochondrial matrix, catalase, glutathione peroxidase and glutathione reductase) and non-enzymatic antioxidants (vitamin A, E and C, carotenoids; beta-karotene and zeaxanthin, as well bioflavonoids, zinc, selene and coenzyme Q) are involved in the protection of the intracellular components against ROS (Moriarty-Craige et al., 2005[82]).

When cellular ROS overproduction overwhelms intrinsic antioxidant capacity then the oxidative stress (OS) occurs as result of either excessive ROS production, mitochondrial dysfunction, impaired antioxidant system or a combination of these factors (Dröge, 2002[26]). The pro-/anti-oxidative cellular imbalance between the ROS production and ability of the biological systems' defense mechanisms to eliminate the cellular stress disturbances leads to the vicious circle, since oxidative stress reciprocally aggravates ROS production. 

Pathological ROS excessiveness causes oxidative damage of the proteins, lipids and nucleic acids. There are several markers of oxidative damage, including: protein carbonyl groups; a marker of protein oxidation, malondialdehyde (MDA) and 4-hydroxynonenal (4-HNE); two markers of lipid peroxidation, 8-hydroxy-2-deoxyguanosine (8-OHdG); a marker of oxidative damage to desoxyrybonucleic acid (DNA) (Cui et al., 2012[23]; Evans et al., 2004[27]; Finkel, 2011[31]). ROS attack structural and enzymatic proteins by the oxidation of residual amino acids, prosthetic groups, formation of cross-links, protein aggregates and proteolysis. The inactivation of the key proteins can lead to the serious consequences in the vital metabolic pathways (Cui et al., 2012[23]; Evans et al., 2004[27]; Finkel, 2011[31]). Lipid peroxidation (auto-oxidation) means oxidation of polyunsaturated fatty acids (PUFAs) due to the presence of several double bonds in their structure and involves production of peroxides (chemical compounds in which two oxygen atoms are linked together by a single covalent bond) (Cui et al., 2012[23]; Evans et al., 2004[27]; Finkel, 2011[31]). ROS react with nucleic acids attacking the nitrogenous bases and the sugar phosphate backbone. Such reactions evoke single- and double-stranded DNA breaks. DNA damage induced by OS may affect the protein-coding region of mitochondrial DNA (mtDNA) and influence oxidative phosphorylation (Cui et al., 2012[23]; Evans et al., 2004[27]; Finkel, 2011[31]; Gredilla, 2011[41]). Mitochondria are the major source of ROS production and their excess induces mitochondrial damage. Human mtDNA is more susceptible to the oxidative damage than its nuclear counterpart, since it is not protected by histones or other associated proteins, it has intronless regions (parts of the gene that do not contain coding information; they have to be spliced out of precursor ribonucleic acid (RNA) to form mature messenger RNA), high transcription rate and a high susceptibility to the oxidative modifications (Sena and Chandel, 2012[104]). 

ROS can be generated at elevated rates under normal aging and in acute or chronic pathophysiological conditions. The aging of the organism is an inevitable process, since the formation of ROS is a result of normal daily cellular metabolism (Cui et al., 2012[23]; Evans et al., 2004[27]; Finkel, 2011[31]; Gredilla, 2011[41]). The amount of accumulated DNA damage increases with the age due to intensified oxidative stress, impairs in the DNA repair system and decreased antioxidant defense (Cui et al., 2012[23]; Evans et al., 2004[27]; Finkel, 2011[31]; Gredilla, 2011[41]). Mitochondrial dysfunction and oxidative stress play an important role in the senescence process, which means irreversible loss of proliferative potential associated with specific morphological and biochemical features, including the senescence-associated secretory phenotype (SASP) (Galluzzi et al., 2018[33]). The pathological ROS excess and OS trigger phenomenon called stress induced premature senescence (SIPS), characterized by the irreversible cessation of the division of normal cells, even in the presence of mitogenic and nutritional factors (Toussaint et al., 2002[118]). SIPS can be stimulated by exogenously administered oxidants like H_2_O_2_ and tert-butyl hydro-peroxide (tBH) (Marazita et al., 2016[73]; Yu et al., 2009[129]), depletion capacity of glutathione (intracellular antioxidant) (Sun et al., 2018[112]) and DNA-damaging mediators (Glotin et al., 2008[39]; Maciel-Baron et al., 2016[72]). Pathological ROS excess, oxidative stress, mitochondrial dysfunction and the cells' inability to repair the incurred mitochondrial damages may lead to DNA mutations and even cell death (Cui et al., 2012[23]; Evans et al., 2004[27]; Finkel, 2011[31]; Gredilla, 2011[41]). Mutations in the key DNA repair genes result in an impaired recognition system and an inefficient repair of DNA damages, which accelerate the aging of the organism. Moreover they lead to the age-related disruptions in cellular and tissue functions, stimulate carcinogenesis, and development of many neurodegenerative diseases (Cui et al., 2012[23]; Evans et al., 2004[27]; Finkel, 2011[31]; Gredilla, 2011[41]). mtDNA mutations cause disturbances in the respiratory chain and the loss of control of ROS production. The much less effective repair system for mtDNA damage may be the cause for accumulating the oxidative stress together with its consequences (Cui et al., 2012[23]; Evans et al., 2004[27]; Finkel, 2011[31]; Gredilla, 2011[41]). Cell death is an irreversible degeneration of vital cellular functions characterized by notably decrease of adenosine triphosphate (ATP) production (an organic high-energy molecule found in every living cell to store and supply the cell with needed energy to drive many vital processes), preservation of redox homeostasis and culminating in the loss of cellular integrity; characterized by permanent plasma membrane permeabilization or cellular fragmentation (Galluzzi et al., 2018[33]). Cell death induced by the excessive ROS production and the oxidative stress is involved in pathomechanism of many general neurodegenerative pathologies such as Alzheimer's disease (Butterfield et al., 2001[15]), Parkinson's disease (Onyango, 2008[89]), prion disease (Kim et al., 2001[63]), protein misfolding diseases (Tabner et al., 2005[114]), age-related macular degeneration and other ophthalmological diseases (Nita and Grzybowski, 2016[84]).

Autophagy is a collective term for the complex lysosomal clearance processes utilized by the cells to eliminate large, unwanted structures to maintain balance between the production of proteins and organelles and their clearance. This strictly regulated genetic process serves for degradation and elimination of no longer needed intracellular components, i.e. cytoplasmic ubiquitinated macromolecules or damaged organelles (mitochondria, endoplasmic reticulum and peroxisomes) (Lee et al., 2012[68]; Mizushima and Komatsu, 2011[80]; Rodrıguez-Muela et al., 2013[97]). Autophagy also acts as a host defence response to many detrimental environmental agents (Blasiak et al., 2014[7]; Labbe and Saleh, 2008[66]); it can be triggered in the infected cells as a host defense mechanism for eliminating the pathogen without disposing of the entire cell (Labbe and Saleh, 2008[66]) and acts as important means for cell survival in conditions of nutritional starvation (during starvation, when the cell literally “eats itself”, autophagy enables recycle of cellular material for maintaining homeostasis of cellular energy, which is reused by recycling amino acids, free fatty acids and nucleotides after their degradation) (Geeraert et al., 2010[35]; Mizushima and Komatsu, 2011[80]). 

The autophagic pathway is activated by numerous stimuli such as: increase in the presence of aggresomes (protein inclusions formed by misfolded proteins aggregated in the perinuclear region), damaged organelles (Bonet-Ponce et al., 2016[10]; Kopito, 2000[65]), depletion of ATP (Kopito, 2000[65]), excessiveness of ROS, oxidative stress and hypoxia (Blasiak et al., 2014[7]; Ferrington et al., 2016[29]; Hariharan et al., 2011[45]; Kaarniranta et al., 2012[60]; Szatmári-Tóth et al., 2016[113]). Inflammation enhances the accumulation of autophagic markers as well (Hariharan et al., 2011[45]; Szatmári-Tóth et al., 2016[113]).

More than 30 autophagy-related genes regulate dynamic autophagy process, which can be divided into induction, nucleation, elongation, fusion and degradation step (Rubinsztein et al., 2012[99]). Autophagy is initiated from omegasome origin when a single-membrane phagophore engulfs the cytosolic contents to form mature, not active double-membrane autophagosome. The autophagosome fuses with lysosomes and forms autolysosome. Lysosomal enzymes are released into autophagosome cavity and start degradation its contents, i.e. aggregated or long-lived proteins, lipid droplets and damaged organelles (Blasiak et al., 2014[7]; Ferrington et al., 2016[29]; Kaarniranta et al., 2012[56]). Many studies and reports describe extensively different aspects of autophagic pathway with regard to receptors and markers of autophagic flux (Ferrington et al., 2016[29]; Hyttinen et al., 2013[51], 2014[50]; Rubinsztein et al., 2012[99]), positive (stimulators) and negative (inhibitors) autophagy regulators (Liu et al., 2016[70]), as well as function of autophagic regulators as a potential target for the development of therapeutics for neurodegenerative diseases including retinal diseases (Cheng et al., 2013[20]; Kaarniranta et al., 2012[56]).

Three different types of autophagic pathways have been investigated. Microautophagy classified as non selective phenomenon occurs under starvation conditions to provide essential nutrients for cell survival (Reggiori et al., 2013[96]). Macroautophagy and chaperone-mediated autophagy are both classified as selective lysosomal process which degrades specific intracellular components, i.e: protein aggregates (aggresomes), stress granules and lipids, cellular organelles such as mitochondria, endoplasmic reticulum (ER), ribosomes and others. The terms aggrephagy (aggregates), mitophagy (mitochondria), granulophagy and lipophagy (stress granules and lipid droplets), reticulophagy (ER), ribophagy (ribosomes) and xenophagy (exogenous pathogens) indicate selective autophagy respectively to the eliminated cargo (Rogov et al., 2014[98]). Selective autophagy utilizes receptors on the autophagosome to bind specific structural elements on the aggresomes or organelles (Boya et., 2013[13]). Recognition and chaperoning of cargo by autophagy receptors to the autophagosome is the initial step of macroautophagy. Following receptor binding, the cargo is chaperoned to the autophagosome where it is degraded by lysosomes (Rogov et al., 2014[98]). 

A proper selective autophagy of aggresomes diminishes cell death (Harris et al., 2011[47]). Mitophagy also acts as cellular pro-survival strategy by which mitochondria are selectively degraded in response to their damage or in response to the hypoxic conditions (Ding and Yin, 2012[24]). Mitochondria are partakers of many autophagic activation stimuli. Failures in mitochondria function not only raise ROS production but also decrease ATP levels (Kopito, 2000[65]). In the healthy cells mitophagic response are present at a basal level to maintain mitochondrial function under changing cellular conditions (Razani et al., 2012[95]). Two dynamic processes, i.e. fission (sequestration away of damaged segments from the mitochondrial network) and fusion of adjacent mitochondria (functional and damaged mitochondria join to complement dysfunctional mitochondria by diffusion and sharing of components between organelles) play crucial role in mitophagic degradation. Fission followed by selective fusion segregate dysfunctional mitochondria and promote their removal by selective autophagy, when cells experience metabolic or environmental stresses. Both processes facilitate the constant remodeling of mitochondrial architecture and serve for ridding the cell of defective mitochondria to maintain homostasis of functional mitochondria (Ban et al., 2010[4]; Pollanck, 2010[93]; Twig et al., 2008[120]). Mitophagy receptors are only negligibly expressed under the normoxic conditions, however they are significantly upregulated under hypoxia (Ferrington et al., 2016[29]). Selective removal of mitochondria under the hypoxic conditions prevents excessive production of ROS by the damaging mitochondria (Ferrington et al., 2016[29]). Mitophagic clearance of dysfunctional mitochondria requires the coordinate action of both the autophagy and proteasome pathways (Ferrington et al., 2016[29]).

With the age markedly decline macroautophagic activity but increases chaperone-mediated autophagy (Rodrıguez-Muela et al., 2013[97]). Deficient autophagy in aging cells results in damaged cell`s viability because of impaired mitophagy, increased ROS generation (Razani et al., 2012[95]), excessive protein oxidation and lipid peroxidation mediated by ROS (McCray and Taylor, 2008[76]), burden in the cytosol of damaged macromolecules and organelles (Dixon at al., 2012[25]), as well as chronic and upregulated inflammation (Ardeljan et al., 2014[2]; Nita et al. 2014[85]; Tseng et al., 2013[119]; Zhou et al., 2009[133]). In many types of cell dysregulation and excessive activation of autophagy lead to lysosomal leakage, cytotoxicity and autophagic cell`s death (ACD). ACD is a non-inflammatory form of cell`s death, since cells undergoing ACD are internalized by the neighboring cells and intracellular, pro-inflammatory content with lysosomes is not spilled to the extracellular matrix (space). Morphological features of ACD include vacuolization, degradation of cytoplasmic content and lack of chromatin condensation (Labbe and Saleh, 2008[66]). Autophagy plays essential role in SIPS and in ferroptosis as well (Sun et al., 2018[112]). Ferroptosis is a form of regulated cell`s death (RCD), initiated by lipid peroxidation (Dixon et al., 2012[25]) and inhibited by iron chelators and lipophilic antioxidants (Galluzzi et al. 2018[33]). It plays significant role in degenerative and neoplastic diseases, and differs from other types of RCD at genetic, biochemical and morphological levels (Dixon et al., 2012[25]).

**Figure 1:**
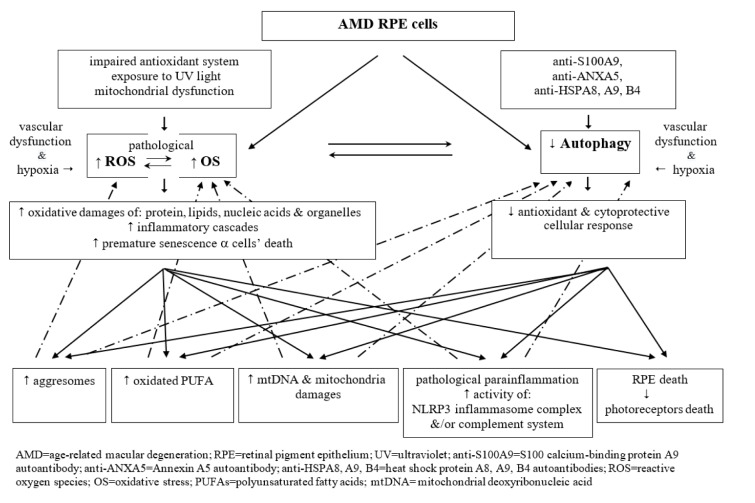
A diagrammatic presentation of disturbed crosstalk between pathological excessiveness of reactive oxygen species and oxidative stress, and dysfunctional (insufficient) autophagy pathway in the aged AMD RPE cells

Figure 1(Fig. 1) presents collectively the interplay between excessiveness of ROS, oxidative stress and faulty autophagy pathway in the course of AMD. 

## Interactions between Reactive Oxygen Species, Oxidative Stress and Autophagy Pathway in the Pathogenesis of the Age-Related Macular Degeneration

### Pathological biomolecules as the source of excessivenes ROS and destabilization of autophagy pathway 

#### Interplay between ROS, lipofuscin/A2E and autophagy

The RPE cells localized between the photoreceptors and the choroid are vitally important for maintaining the viability of the photoreceptors and retinal homeostasis. RPE absorbs light, acts as a retinal blood barrier and delivers blood-derived nutrients to photoreceptors, transports water, ions and metabolic products from the subretinal space to the blood, releases polarized growth factors, and performs phagocytosis of the photoreceptors' outer segments (POS) (Strauss, 2005[111]). The RPE cells are phagocytically the most active cells in the whole body, uptaking and degrading up to 10 % of POS daily (Strauss, 2005[111]). Daily efficient heterophagy (the digestion within cell of material ingested via phagocytosis or pinocytosis) of POS and their degradation in RPE lysosomal system is necessary to keep up normal photoreceptors function and to support retinal homeostasis (Kaarniranta et al., 2018[60]; Kim et al., 2013[64]; Zhang et al., 2015[131]). The apical microvilli of the RPE cells extend around POS and ingest rod and cone outer segment discs into RPE as membrane-bound phagosomes, which subsequently fuse with RPE lysosomes to form phagolysosomes (Kaarniranta et al., 2018[60]; Kim et al., 2013[64]). After the fusion of lysosomes and autophagosomes, the substrates for macroautophagy are degraded in the RPE cells by lysosomal acid hydrolases, including cathepsins D, B, and L. Rab7, LAMP-2A, and SNAREs proteins are critical for the lysosome and autophagosome fusion process, however, fusion mechanisms in the RPE cells are under investigation. Ubiquitin (Ub), LC3II, and p62 are the elements of cargo and they connect autophagy to the proteasomal clearance system (Blasiak et al., 2014[7]). RPE lysosomal acid hydrolases degrade POS material which is reused in the photoreceptors, and this degradation process is critically controlled by acidification of lysosomes with vacuolar-type H + -ATPase (V-ATPase) (Kim et al., 2013[64]; Valapala et al., 2014[121]). Efficient RPE lysosomes activity is critical for sustaining normal retinoid levels and visual cycle (Kaarniranta et al., 2013[58], 2018[60]; Kim et al., 2013[64]; Valapala et al., 2014[121]). It is widely accepted that defects in phagocytosis are detrimental to the RPE cells and photoreceptors (Blasiak et al., 2014[7]; Vives-Bauza et al., 2008[122]). 

Phagocytozed POS, highly enriched in polyunsaturated fatty acids (PUFAs), are the major source of intracellular ROS generation in RPE cells (Cai et al, 2000[16]), however, high partial oxygen pressure in RPE is also due to anatomical proximity to choriocapillaries (Khandhadia and Lotery, 2010[62]). The age-related imbalance between ROS production and antioxidant defense responses, such as catalase and superoxide dismutase activity, results in overproduction of ROS and enhancement of oxidative stress in the RPE cells (and the whole retina), which with time damage RPE tight junctions and disrupt the retinal blood barrier (Sun et al., 2018[112]), cause molecular and cellular damage of the PRE cells and photoreceptors, lead to intracellular accumulation of detrimental products, stimulate formation of extracellular deposits (drusen); a hallmark of AMD (Jong, 2006[55]), and accelerate AMD development (Sun et al., 2018[112]). Increased ROS activity and disturbed (decreased) lysosomal activity of autophagy pathway in the RPE cells along with the growth of age lead to insufficient digestion of oxidated PUFAs and to their accumulation in RPE lysosomes and cell membranes in the form of lipofuscin (Vives-Bauza et al., 2008[122]). Products of lipid peroxidation can also increase lipofuscin accumulation and reduce activity of RPE autophagy (Zhang et al., 2015[131]). 

Lipofuscin is a heterogenous protein-lipid-carbohydrate aggregate, which includes toxic fluorophores such as N-retinylidene-N-retinylethanolamine (A2E) and its photoisomers (Vives-Bauza et al., 2008[122]). The RPE cells are constantly exposed to irradiation and to light-induced oxidative stress stimulated by UV and blue light (Cai et al., 2000[16]). Lipo-fuscin acts as the main RPE photosensitizer (Schutt et al., 2002[103]; Sparrow et al., 2002[107]). After absorbing a high-energy photon; especially that of blue light (from 450 to 495 nm), A2E undergoes a variety of photochemical reactions, which increase ROS generation, accelerate oxidative stress in RPE mitochondria, inhibit mitochondrial respiration and in turn evoke lipid peroxidation in cell membrane (Sparrow et al., 2002[107]; Vives-Bauza et al., 2008[122]). 

Photooxidation of A2E accumulated in RPE lysosomes is responsible for RPE lysosomal membrane destabilization and has properties to disrupt other cellular membranes (Schutt et al., 2002[103]). 

A2E acts as strong autophagy pathway stimulator. After only 15 minutes of incubation with A2E (25 μM), the RPE cells initiated formation of autophagosomes and after 6 hours autophagosomes started to fuse with lysosomes to form autophagolysosomes. The course of autophagy in A2E-treated RPE cells confirmed the buildup of a punctate pattern of cytosolic LC3 and the upregulation of LC3-II and Beclin-1; all those are the important markers of autophagosome formation (Zhang et al., 2015[131]). mTOR regulates the detrimental dedifferentiation and hypertrophy of RPE cells that have been exposed to the oxidative stress, whereas treatment with rapamycin can prevent these effects and preserve photoreceptor function (Zhao et al., 2011[132]). Rapamycin alleviates also choroidal neovascularization by inhibiting the function of VEGF-A (Stahl et al., 2008[110]). It was shown that both A2E and rapamycin act as autophagy activators through the Akt/mTOR pathway; A2E decreased the expression of p-Akt and p-mTOR, and rapamycin inhibited the Akt/mTOR signaling pathway in A2E-treated RPE cells as well. Moreover, rapamycin improved protective effect on the RPE cells against A2E by inhibiting the inflammatory response and reducing function of angiogenic cytokine VEGF-A. All these findings indicate that autophagy intervention will be valuable in preventing AMD development and progression (Zhang et al., 2015[131]). 

A2E fluorophore has potency to activate the RPE cells to secretion of several inflammatory factors and angiogenic cytokines (Zhang et al., 2015[131]). Chronic inflammation in the retina acts as a strong stimulator of autophagy pathway as well (Ardeljan et al., 2014[2]). A2E RPE lysosomal destabilization stimulates pathophysiological parainflammation in the retina, inducing hyperactivity of the complement system (Nita et al., 2014[85]; Zhou et al., 2009[133]) and the NLRP3 inflammasome complex (Nita et al., 2014[85]; Tseng et al., 2013[119]). Upregulation of these both strong pro-inflammatory mechanisms contribute to GA/AMD and CNV/AMD development via cytokine release and pyroptotic RPE cell death, respectively (Ardeljan et al., 2014[2]). ARPE-19 cells exposed to interleukin-17 (IL-17) show nuclear disintegration in addition to autophagy. Cytosolic DNA from the damaged cellular nuclei is a potent damage-associated molecular pattern (DAMP); also known as danger-associated molecular patterns, danger signals and alarmin, which can initiate and perpetuate a noninfectious inflammatory response (Ardeljan et al., 2014[2]). 

Macroautophagy is prevented in the aged RPE cells, since lysosomal lipofuscin disturbs cathepsin activity and autophagy flux (Blasiak et al., 2014[7]). Dysregulated or declined autophagy pathway increases oxidative stress, enhances photochemical degeneration of RPE, evokes lipofuscin accumulation and protein aggregation, and accelerates AMD progression (Kaarniranta et al., 2013[58]; Szatmári-Tóth et al., 2016[113]; Vives-Bauza et al., 2008[122]; Wang et al., 2009[123]). Disturbed lysosomal clearance in the aged and degenerated RPE cells can be clinically observed by pigment mottling, increased autofluorescence and accumulation of extracellular drusen deposits. All those are hallmarks of increased RPE cellular stress and degeneration, which over the time may lead to RPE cell death (Kaarniranta et al., 2017[59]). 

#### Interplay between methylglyoxal-modified proteins and autophagy 

Methylglyoxal (MGO)-modified proteins are major precursors of advanced glycation end (AGE) products (Yoon et al., 2012[128]). AGEs arise from non-enzymatic glycation or glycooxidated after the exposure to the aldose sugars nucleic acids, proteins and lipids (Schmidt et al., 2000[102]). Normal AGEs serum level in the range of 2-10 Lg/ml (Yamagishi et al., 2007[126]) increases in the course of physiological aging, which is caused by the imbalance between increasing oxidant production and decreasing antioxidant capacity, and leads to the accumulation of this soluble and protease-resistant aggregates (Schmidt et al., 2000[102]). Accumulated intra- and extracellulary, nondegradable AGEs impair directly and indirectly normal cellular structure and function after binding to the specific receptors for advanced glycation end products (RAGEs) (Schmidt et al., 2000[102]). AGEs/RAGEs pathway results in the apoptotic cell death, cell activation and proliferation, angiogenesis, chemotaxis and cause loss of many protein functions, including their involvement in the regulation of gene transcription (Schmidt et al., 2000[102]). AGEs are a hallmark of chronic neurodegenerative disease (Alzheimer's disease (Lue et al., 2005[71])) and others pathologies (atherosclerosis (Harja et al., 2008[46]) and diabetes (Chilelli et al., 2013[21]), and represent a risk factor of AMD development as well (McCarty et al., 2001[75]; Yoon et al., 2012[128]). AGEs are endocytozed and removed by macrophages (Ohgami et al., 2001[88]). The failure in their recrutation leads to increased RPE exposure with AGEs and damage retinal tissue in the course of chronic, pathophysiological parainflammation (Lin et al., 2013[69]; Nita and Grzybowski, 2014[84]). Laboratory studies confirmed AGEs deposits in soft drusen (Crabb et al., 2002[22]) and in Bruch's membrane (Tian et al., 2005[117]). AGEs promote oxidative stress, lipofuscin accumulation, stimulate RPE cells to secretion of various complex pro- and anti-inflammatory factors and cause apoptotic RPE cell death in the aged retina (Glenn et al., 2009[38]). Formation and metabolism of methylglyoxal (MGO)-modified proteins, AGEs and AGEs-like adducts can induce cellular oxidative stress injury (Yoon et al., 2012[128]). 

It was shown that MGO concentrations higher than 125 µM significantly increased ROS production, aggravated oxidative stress, potentiated H_2_O_2_-induced autophagy flux and H_2_O_2_-induced cytotoxicity in ARPE-19 cells exposed to 250 µM H_2_O_2_, a positive agent for autophagic induction. MGO significantly upregulated levels of LC3 I and LC3 II proteins (LC3 II is a marker of autophagosome maturation) in such doses. Increased intracellular accumulation of autophagosomes in ARPE-19 cells was also confirmed functionally by addition of autophagy enhancer or inhibitor. MGO remarkably triggered phosphorylation of protein kinase B; also known as Akt, extracellular signal-regulated kinase 1/2 (ERK1/2), p38 mitogen activated protein kinase (p38 MAPK), and c-Jun NH2-terminal kinase 1/2 (JNK1/2). Blockade of kinase activity demonstrated that the hyperphosphorylation of Akt, ERK1/2, p38 MAPK and JNK1/2 were all involved in the MGO-enhanced autophagy and growth-arresting effect in ARPE-19 cells. However, pretreatment with autophagic flux inhibitors including 3-methyladenine, bafilomycin A, and chloroquine effectively ameliorated MGO, but not H_2_O_2_-mediated ARPE-19 cytotoxicity. The authors stated that modulation of autophagy flux activity by using autophagic or kinase inhibitors may be an applicable modality to treat AMD (Chang et al., 2015[19]).

#### Misfolding protein deposits and autophagy-mitochondrial-endoplasmic reticulum crosstalk

To maintain proteostasis and cell function, the endoplasmic reticulum (ER) of the RPE cells activates an adaptive quality control measure known as the unfolded protein response (UPR), initiated by three independent transmembrane stress transducers, i.e.: inositol-requiring kinase-1 (IRE1), double-stranded RNA-activated protein kinase-like ER kinase (PERK), and activating transcription factor-6 (ATF6) (Hamasaki et al., 2013[44]; Matsunaga et al., 2016[74]; Minasyan et al., 2017[79]). Aggrephagy is responsible for the clearance of misfolding protein deposits (Yamamoto and Simonsen, 2010[127]). Mitochondria and ER contact sites are involved in the autophagosome formation, and many proteins in the mitochondria-associated ER membrane (MAM) compartments are necessary for the autophagic vesicle formation (Hamasaki et al., 2013[44]). 

Autophagy-mitochondrial-ER crosstalk plays a role in homeostasis of the RPE cells (Sreekumar et al., 2016[109]). Lipofuscin inhibits mitochondrial respiration and increases ER stress in RPE cells (Feng et al., 2014[28]; Nordgaard et al., 2008[87]). Decline in autophagy-mitochondrial-ER clearance system in RPE leads to the increased mitochondrial-ER stress, oxidative stress and inflammation, and favors AMD development (Sreekumar et al., 2016[109]). The mitochondrial-derived peptide humanin; a prominent member of a newly discovered family of mitochondrial-derived peptides expressed from mitochondrial 16S rRNA, protects the RPE cells from oxidative stress, senescence, and mitochondrial dysfunction (Sreekumar et al., 2016[109]). Humanin G, the more potent variant of humanin, protects the RPE mitochondria and ER together with reducing the apoptotic cell death at both gene and protein levels (Sreekumar et al., 2016[109]). The therapeutic use of humanin could potentially prove to be a valuable approach for AMD treatment (Kaarniranta et al., 2013[58]).

### Mitochondrial dysfunction and mtDNA damages as the source of excessiveness ROS and autophagic stimulators

Aging results not only in shortage of antioxidant defense and increased sensitivity to oxidative stress but it also reduces RPE mitochondrial membrane potential (bioenergetics deficit) (He et al., 2010[48]), and stimulates senescence-associated mitochondrial dysfunction in the RPE cells (Wiley et al., 2016[125]). In human RPE the number of mitochondria decreases with the age, however their length increases, and this elongation results from oxidative/metabolic stress (He et al., 2010[48]). Weakened antioxidant protection combined with the shortage of mitochondrial capacity strongly reduces RPE function and contributes to the AMD onset (Hyttinen et al., 2018[53]). 

Non-invasive functional retinal imaging technique, called flavoprotein fluorescence (FPF), revealed that elongated and dysfunctional mitochondria form parallel clusters oriented perpendicularly to the basal membrane of the RPE cell in GA/AMD. The heterogeneity of FPF images indicates increased variability in the severity of the damages in the eyes observed inadvanced AMD (Field et al., 2012[30]). Laboratory studies of human RPE AMD donors confirmed decreased numbers of mitochondria and showed additionally disorganization and loss of mitochondria cristae (the folds in the inner mitochondrial membrane), reduction of the mitochondrial matrix density, defective fission/fusion processes, lower levels of mitochondrial respiratory chain proteins, and mutations in mtDNA (Karunadharma et al., 2010[61]; Terluk et al., 2015[116]). The age-related dysfunctional RPE mitochondria become a source of excessively accumulated ROS and damages of mtDNA (Hyttinen et al., 2017[52]; Karunadharma et al., 2010[61]; Terluk et al., 2015[116]). mtDNA damages occur already in the early phases of AMD and they are limited to RPE, in both the macular and peripheral regions (Terluk et al., 2015[116]).

Mitochondria may stimulate general autophagy, and ROS-dependent destructive mechanisms underline this effect. Mitochondria are targeted by mitophagy, which should be considered not only as a type of macroautophagy but mainly as a crucial mechanism that manages mitochondrial quality control in stress situations, to prevent the accumulation of damaged mitochondria and to protect RPE cells against oxidative stress (Hyttinen et al. 2018[53]). Mitophagy seems to be independent of the general autophagy in the regard of senescence (Hyttinen et al. 2018[53]), however, its activity is reduced in the aged cells (García-Prat et al., 2016[34]; Hyttinen et al., 2018[53]). Mitophagy is activated later in the aged RPE cells since mTORC1 activity is elevated in aged cells (Hyttinen et al., 2018[53]). Lowered mitophagy in the aged RPE cells may cause PINK1 deficiency, which suppresses mitochondrial fission and elimination of dysfunctional parts of the mitochondrial “network” (Bueno et al., 2015[14]; Hyttinen et al., 2018[53]). Mitophagy can be also inhibited in aged RPE cells by p53 protein, which interacts with Parkin and prevents its localization on the damaged mitochondria (Ahmad et al., 2015[1]; Hyttinen et al., 2018[53]). 

Dynamic modeling of mitophagic and cellular senescence pathways reveals strategies for targeted interventions in AMD (Pezze et al., 2014[92]) and the suppression of PINK 1 dficiency may be one of such therapeutic strategies (Hyttinen et al., 2018[53]). AICAR (5-aminoimidazole- 4-carboxamide-1-β-ribofuranoside) enhances the efficacy of rapamycin, which acts as mTOR supressor (Palikaras et al., 2017[90]). AMP activated protein kinase (AMPK) increases activity of autophagy/mitophagy pathway. AMPK can be induced by metformin, a diabetes type 2 drug in use (Palikaras et al., 2017[90]). Metformin induces also MRC complex I function (Foretz et al., 2014[32]), activates mitophagy by reducing the abundance of cytosolic tumor suppressor factor p53, which act as mitophagic inhibitor via interaction with Parkin (Song et al., 2016[106]), metformin blocks pro-inflammatory NF-kappa B (NF-ĸB) signaling (Saisho, 2015[100]), and inhibits the secretory-associated senescence phenotype (Moiseeva et al., 2103[81]). To sum up, metformin may act as a senostatic drug to inhibit the senescent phenotype in AMD (Hyttinen et al., 2018[53]).

Mitochondrial biogenesis and autophagy/ mitophagy process can be enhanced by inducing peroxisome proliferator-activated receptor gamma-coactivator-1α (PGC-1α) via silent information regulator 1 (Sirt1) (Cantó et al., 2009[17]; Palikaras et al., 2017[90]). Resveratrol, a polyphenolic natural compound, stimulates Sirt1 indirectly by elevating NAD+ levels and by activating AMPK (Morselli et al., 2010[83]). PGC-1α , Sirt1 and AMPK represent new therapeutic targets for interventions in AMD (Cantó et al., 2009[17]). Efficient mitophagy can prevent RPE cell death. Early intervention in mitochondrial quality control to prevent DNA damages may be useful in the attenuation of AMD progression. This could be done via modulation of mitophagic activity when mitochondria with damaged DNA and disturbed expression of important proteins involved in mitochondrial respiratory chain could be removed (Hyttinen et al., 2018[53]).

Studies confirm alterations in iron homeostasis and increased iron accumulation in AMD retinas, which contribute to the excess ROS formation catalyzed by Fe3+ ions, increased expression of the Divalent metal transporter 1 (DMT 1) and the decrease of Ferroportin 1 (FPN 1) gene expressions (Blasiak et al., 2011[8]). In neurodegenerative diseases, dysfunctional mitochondria show also changes in iron homeostasis, and if it happens in the secluded space of mitochondria it leads to the decrease of MRC complex I activity (Mena et al., 2015[77]). Mitochondrial ferritin (FtMt) is an iron-storage protein, which controls antioxidant capacity via regulation of Fe storage and plays role in AMD pathophysiology (Wang et al., 2016[124]). Human ARPE-19 cells showed FtMt overexpression, which stabilized HIF-1α but increased VEGF secretion, and conversely, HIF-1α stabilization reduced the mature, functional form of FtMt. FtMt-overexpressing ARPE-19 cells exhibited less oxidative phosphorylation but unchanged production of adenosine triphosphate, enhanced mitochondrial fission, and triggered mitophagy in a HIF-1α-dependent manner. These findings suggest dual role of increased levels of FtMt in AMD RPE cells; protective influence via triggering mitophagy but unfortunately detrimental influence via inducing increased VEGF secretion leading to CNV/AMD. Moreover, reduced level of mature FtMt may facilitate GA/AMD under hypoxia conditions through susceptibility to the age-related stress (Wang et al., 2016[124]). 

With the age, the reduced capacity to “clean” the damaged mitochondria and the failure of mitophagy trigger inflammation via inflammasome activity (Salminen et al., 2012[101]). Inflammasome is a high molecular weight multiprotein cytosolic assembly composed of receptors and sensors, stimulated by cellular stress, tissue damage, pathogens or infection. This key mediator engaged to innate immune system responses activates caspase-1 by facilitating the cleavage of its zymogen (an inactive precursor), i.e. pro-caspase-1. Active caspase-1 activates proteolytic maturation of two pro-inflammatory cytokines, i.e. interleukin 1beta (IL-1β) and interleukin 18 (IL-18). Both interleukins are synthesized as the inactive cytosolic precursors, i.e. pro-IL-1β and pro-IL-18 (Bauernfeind et al., 2011[5]). The cell requires priming via NF-ĸB signaling to downstream IL-1β expression, and any molecule which activates NF-ĸB is a potential priming agent (Hiscott et al., 1993[49]). As resulting from lysosomal destabilization, inflammasome mediates caspase-1 dependent cytotoxicity named “pyroptosis”, which is pro-inflammatory mode of regulated cell death characterized by plasma membrane rupture and release of intracellular contents (Bergsbaken et al., 2009[6]; Galluzzi et al., 2018[33]).

The RPE cells actively participate in the host's immune defense responses via expression of NLRP3 inflammasome complex to maintain homeostasis of photoreceptors and the outer retina (Tseng et al., 2013[119]). However, RPE dysfunction and drusen formation with the age generate a pro-inflammatory milieu in the RPE-Bruch's membrane interface, which stimulates chronic, pathological hyperactivity of NLRP3 inflammasome pathway (Hageman et al., 2001[43]; Tarallo et al., 2012[115]). It was confirmed that up-regulation of NLRP3 protein in the RPE cells of donor human eyes affected by both GA/AMD and CNV/AMD (but not in eyes of age-matched controls). NLRP3 staining was detected at lesion sites intracellularly in the RPE cells and extracellularly in soft drusen, as well as in the vicinity of Bruch's membrane (Tseng et al., 2013[119]). The authors showed, that NLRP3 inflammasome activation and its downstream effects are induced by lysosomal destabilization, since chemically induced lysosomal disruption by L-leucyl-L-leucine methyl ester (Leu-Leu-OMe) triggered caspase-1 activation, IL-1β release and pyroptotic death of the RPE cells, however, its blocking with Gly-Phe-CHN2 (it inhibits the DPP-I-dependent conversion of Leu-Leu-OMe to a membranolytic derivative inside the lysosome) or inhibiting caspase-1 with Z-YVAD-FMK abrogated IL-1β release and pyroptotic RPE cell death (Ardeljan et al., 2014[2]). They also presented a two-signal model of NLRP3 inflammasome induction in AMD. IL-1α, tumor necrosis factor alpha (TNFα) and adduct of carboxypyrrole product (CEP) are priming signals 1, which activate NF-ĸB to induce expression of NLRP3 and pro-IL-1β. Inflammasome activates caspase-1, which cleaves pro-IL-1β to form mature IL-1β and mediates primed pyroptotic RPE cell death. Lipofuscin, components of soft drusen and other insoluble lysosomal contents belongs to priming signals 2, which activates NLRP3 inflammasome via lysosomal enzymes such as cathepsins B and L (Tseng et al., 2013[119]). Accumulation of Alu RNA (a modular transacting repressor of mRNA transcription during heat shock) due to DICER1 gene deficiency induces GA/AMD (Tarallo et al., 2012[115]). Alu RNA cytotoxicity or DICER1 deficit activates the NLRP3 inflammasome in the RPE cells, and triggers toll-like receptor independent MyD88 (a central player in innate immune signaling) via IL-18 (Tarallo et al., 2012[115]). Genetic or pharmacological inhibition of inflammasome components (NLRP3, Pycard, Caspase-1), MyD88, or IL-18 may prevent RPE degeneration induced by Alu RNA exposure or DICER1 loss (Tarallo et al., 2012[115]). 

### Vascular dysfunction as the source of ROS and faulty autophagy in AMD

#### Interplay between hypoxia and autophagy 

Negative properties of ROS may affect vascular molecules, disturbing choroidal and retinal blood flow (Boltz et al., 2010[9]). Vascular dysfunctions lead to overproduction of ROS and oxidative stress stimulation, which within vicious circle induce changes in the choroidal and retinal vessels (Kaarniranta et al., 2009[57]). Oxidative stress is linked to hypoxia in AMD (Blasiak et al., 2014[7]). Hypoxia is defined as a decrease in available oxygen reaching the tissues of the body. Decline of oxygen concentration below the range from 3 to 5 % is usually considered as hypoxia (Metelitsina et al., 2008[78]). In the retina, hypoxia is a result of diminished choroidal blood circulation (Grunwald et al., 2005[42]; Metelitsina et al., 2008[78]). The reduction in choroidal perfusion has been positively correlated with AMD progression (Arjamaa et al., 2009[3]). Retinal blood flow is disturbed in both advanced AMD type, i.e. GA/AMD and CNV/AMD (Grunwald et al., 2005[42]; Metelitsina et al., 2008[78]). Hypoxia stimulates synthesis and release of hypoxia- inducible factor-1 (HIF-1) as well as vascular endothelial growth factor (VEGF) contributing to CNV/AMD development (Kaarniranta et al., 2009[57]). In AMD exist relationships between oxidative stress and deficient autophagy as well as between hypoxia and faulty autophagy. The triplet composed of oxidative stress-hypoxia-autophagy represents a self-fueling chain reaction of events, which accelerates AMD progression (Blasiak et al., 2014[7]).

#### The role of circulating autoantibodies against autophagy regulators in AMD

S100 calcium-binding protein A9 (S100A9), also known as calgranulin B, induces autophagy (and apoptosis) via a ROS-mediated cross-talk between lysosomes and mitochondria (Ghavami et al., 2010[36]), as well as plays a prominent role in the regulation of inflammatory processes and immune response (Skeie and Mahajan, 2014[105]). Elevated expression of S100 proteins showed BrM/ choriocapillares complex of human AMD retinas as compared with normal eyes (Yuan et al., 2010[130]). 

Annexin A5 (ANXA5), an autophagy stimulator (Ghislat et al., 2012[37]), was documented in BrM and drusen (Rayborn et al., 2006[94]), and was upregulated in CNV/AMD tissues as well (Lederman et al., 2010[67]). 

The heat shock protein 70 (HSP70) family is a key effector of chaperone mediated autophagy in RPE cells (Kaarniranta et al., 2009[57]). Disruption of HSPs proteolytic pathway in the aging RPE implicates dysregulation of autophagy, accumulation of oxidative stress-induced damage, protein aggregation, lipofuscinogenesis, and AMD development (Kaarniranta et al., 2009[57]). HSP70s act also as direct downregulators of the inflammatory response mediated by dendritic cells and other innate immune system cells (Borges et al., 2012[11]). 

Circulating autoantibodies (AAbs) directed against targets implicated in autophagy control, i.e.: anti-S100A9 AAbs, anti-ANXA5 AAbs, and anti-HSPA8, A9 and B4 AAbs, can contribute to AMD pathogenesis directly, compromising an autophagy-mediated mechanism, and indirectly, activating downstream of the inflammasomes (Iannaccone et al., 2015[54]).

## Conclusions

Interplay between oxidative stress and autophagy plays an important role in the pathogenesis of the age-related macular degeneration. Disturbed crosstalk between excessiveness of reactive oxygen species and dysfunctional autophagy acts as strong pro-inflammatory factor and pro-senescence mechanism, which stimulates cell death via non-inflammatory autophagic manner and via pro-inflammatory pyroptotic manner. Dynamic modeling of autophagy pathway is one of the new therapeutic strategies in the age-related macular degeneration.

## Funding

Self-financing unit.

## Conflict of interest

The authors declare that there is no conflict of interest regarding the publication of this paper.
